# In search of *LncDACH1* – a perceived intronic long non-coding RNA lacking fundamental genomic annotation evidence

**DOI:** 10.1038/s41467-026-76991-6

**Published:** 2026-08-21

**Authors:** Matthew Bennett, Stan Muitjens, Igor Ulitsky, Andrew H. Baker

**Affiliations:** 1 Centre for Cardiovascular Science, Queen’s Medical Research Institute, University of Edinburgh, Edinburgh, UK; 2 Department of Pathology and Cardiovascular Research Institute Maastricht (CARIM), Maastricht University Medical Centre, Maastricht, The Netherlands; 3 Department of Immunology and Regenerative Biology, Weizmann Institute of Science, Rehovot, Israel

**Keywords:** Long non-coding RNAs, Sequence annotation, Next-generation sequencing, Microarrays

**arising from** Z. Li et al. *Nature Communications*, 10.1038/s41467-024-48019-4 (2024)

The paper “LncRNA-*LncDACH1* mediated phenotypic switching of smooth muscle cells during neointimal hyperplasia in male arteriovenous fistulas” by Li et al., published in 2024 in this journal, proposes that a relatively short unspliced long non-coding RNA (lncRNA) positioned within the intron of the protein-coding gene *DACH1* plays a causal role in the vascular remodelling that often occurs after arteriovenous fistula formation^1^. This is a surgical procedure for patients undergoing haemodialysis but is very often hampered by vascular malformations (stenosis and thrombosis) that result in critical failure of the fistula^2^. We have reviewed this and four prior studies^3–6^ concerning the proposed gene *lncDACH1*. We believe that fundamental questions on the annotation state of the proposed lncRNA gene have not been addressed. We find no evidence presented in the recent study or prior work that suggests the sequence targeted in experiments is a lncRNA—or indeed anything other than a part sequence of *DACH1* pre-mRNA.

The characterisation and study of lncRNAs relies on accurate and robust identification of the lncRNA. In the age of transcriptomics, this relies on a robust signal from confidently aligned RNA-seq reads that are expected to clearly arise specifically from the interrogated gene. The field contains several examples where studies proposing novel lncRNA functions turn out to be targeting artifacts (examples in ref. ^7^ pg.3). Loci where the annotated lncRNA is not spliced and overlaps an intron of another gene transcribed on the same strand require particular rigor, as any transcriptional signal in such loci can arise from either a bona fide lncRNA or from the intron-containing pre-mRNA of the protein-coding gene. Intronic lncRNAs require unambiguous evidence to show they are a distinct transcriptional unit with independent transcriptional initiation and termination. Unspliced lncRNAs are particularly challenging to annotate as they do not contain the more specific splice-junction–spanning reads, and indeed, several lncRNA annotation efforts have either completely excluded unspliced transcripts^8^ or required definitive evidence for unspliced transcripts, excluding those overlapping with other genes^9,10^. As *lncDACH1* completely overlaps an intron of the *DACH1* pre-mRNA and is unspliced, vigilance in study design would be expected to claim that experiments are targeting a distinct functional unit and not simply *DACH1* pre-mRNA. This is a null hypothesis which we believe has not been addressed to any satisfactory degree in this or previous *lncDACH1* papers.

The original *lncDACH1* implication in cardiac biology comes from a 2014 microarray analysis^6^. This short paper reports a highly excessive fraction of intronic lncRNAs (55%) relative to more stringent concurrent lncRNA annotations (GENCODE M19 2.5% https://www.gencodegenes.org/mouse/stats_M19.html). This suggests the analysis was inflated with RNA signals from ambiguous origins. The datasets used for the generation of microarrays at that time (obtained years before the wide adoption of RNA-seq) contained transcripts based on limited experimental support. Therefore, rigorous characterisation of *lncDACH1* and validation that it is indeed an independent transcript and not a fragment of the *DACH1* pre-mRNA should have been obtained in subsequent studies. This could entail a range of techniques such as RACE (to delineate the full 5’ and 3’ of the transcript), RNAseq (to show enriched read coverage at the locus relative to the rest of the intron), FISH (to demonstrate distinct localisation relative to other parts of *DACH1*), CAGEseq (to identify a matching 5’ TSS), or co-expression analysis (to show that *lncDACH1* has a distinct expression pattern to host mRNA or pre-mRNA). Ideally a combination of these approaches would be used to form a robust body of evidence. The widespread availability of RNAseq data, particularly long-read RNAseq collected by PacBio or Nanopore technology, means that beginning this process is well-facilitated for researchers^7,11^. However, no sufficient case for the existence of *lncDACH1* from such techniques has been made in any of the papers. Strikingly, *lncDACH1* is proposed to derive from *DACH1* pre-mRNA and so would share 100% sequence homology with it. However, *DACH1* is not mentioned at any point in this study – and barely in previous studies. Lack of addressing this fundamental question means perturbations performed across all papers – including plasmid overexpression, siRNA treatment, and conditional knockouts in mice may, in fact, be targeting *DACH1* pre-mRNA. Indeed, in rare instances where the effects of *lncDACH1* perturbation on *DACH1* expression level have been shown (in Fig. S10 of Cai et al. 2019^3^ and Fig. S8 of Cai et al. 2020^5^), the analysis appears insufficiently powered to make a conclusive judgement. The low rigor applied in the original study to assignation of a lncRNA thus propagated through all the subsequent studies with limited and insufficient further scrutiny.

We have experience in characterising vascular lncRNAs. Therein, we analysed total RNAseq (including non-polyA transcripts) of relevant samples that match those assayed in this and previous papers (mouse heart, human coronary artery and aortic smooth muscle cells (SMC) from ENCODE read alignments and quiesced saphenous vein SMCs alignments from prior work^12^) to examine if any area of the *DACH1* intron contained an area enriched with read coverage that would be expected if *lncDACH1* indeed existed as an independent transcript. We found no signal at the *lncDACH1* locus that was elevated beyond low-level intronic read mapping across the entire intron (Fig. 1a, b). We also find no robust CAGEseq hits within the *DACH1* intron that would suggest a promoter in this region (Fig. 1c). In contrast, FISH of *lncDACH1* in Fig. 1i of Li et al. 2024^1^ (and in Fig. S4 of Cai et al. 2020^5^) shows an intense cytoplasmic signal, suggesting a highly expressed and exported transcript that would produce a large RNAseq signal in heart or venous SMC. This high expression level is inconsistent with the lack of signal across multiple relevant samples in our RNAseq analysis (we could not find the sequence for the *lncDACH1* FISH probe in this or prior publications). In addition, we note a potential *DACH1* exon overlapping the suggested *lncDACH1* site in the FANTOM CAT reference annotation (Fig. 1c, exon present in transcript FTMT24900018940.1, not seen in GENCODEv47), further underscoring the need for better locus annotation to address this additional ambiguity on the independence of *lncDACH1* from *DACH1*. In summary, we cannot yet identify any evidence that would support the existence of an independent *lncDACH1* transcript in humans or mouse. We believe the null hypothesis that *lncDACH1* signals are all merely a reflection of *DACH1* expression, and that methods used for targeting *lncDACH1* that influence *DACH1* by targeting its intron should be comprehensively addressed by the authors.

**Fig. 1:**
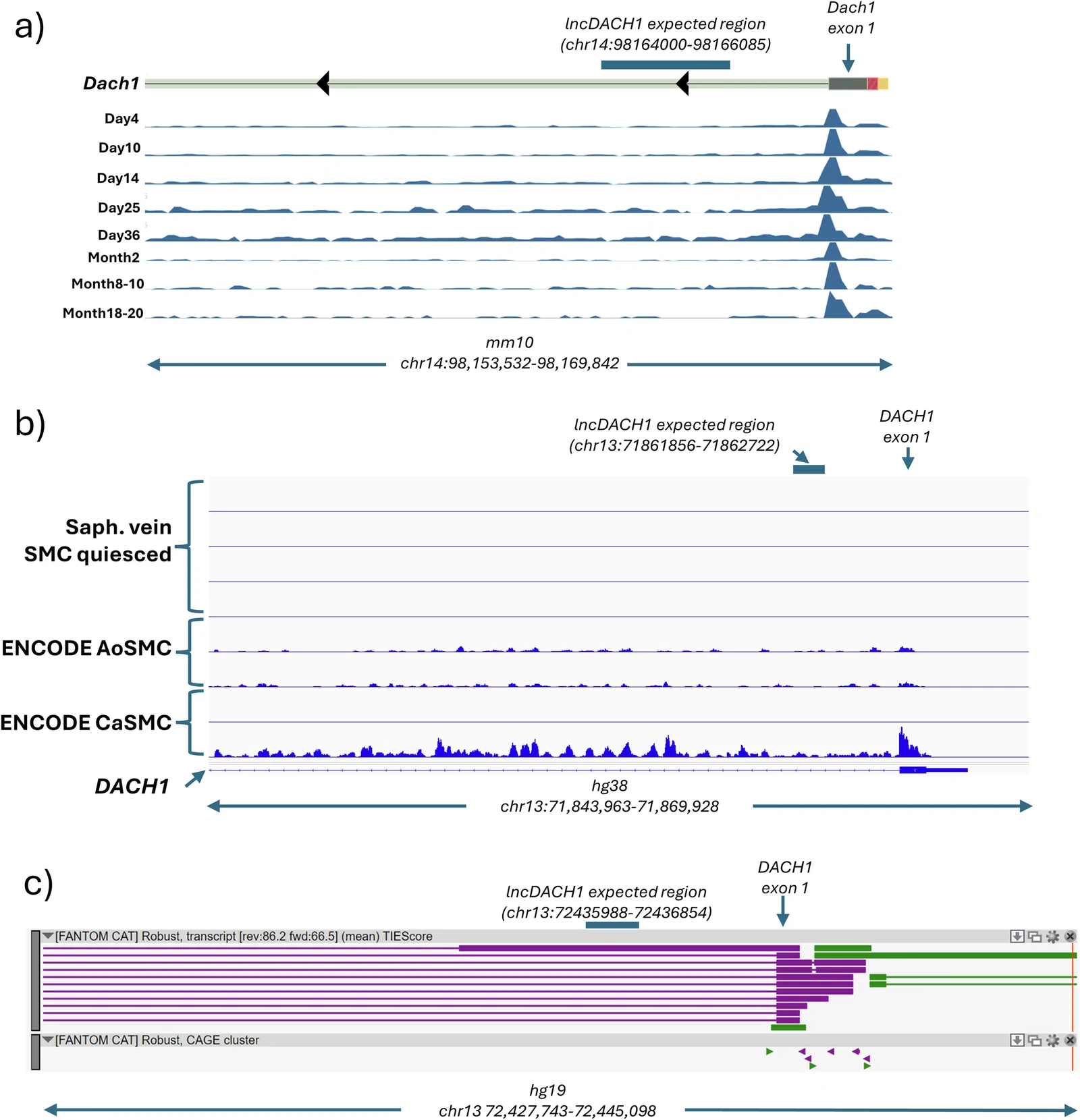
Searching for evidence for the transcription of *lncDACH1* in relevant transcriptomics data. **a** *LncDACH1* is proposed to be expressed in baseline mouse cardiomyocytes and the heart. Expression over the *DACH1* intron in a mouse heart development RNAseq timecourse is shown (**b**) *LncDACH1* is proposed to be expressed in baseline venous smooth muscle cells. Expression over the *DACH1* intron in a panel of human smooth muscle cells available to us. (Ao = aortic, Ca = coronary artery, Saph. vein = saphenous vein). **c** Annotation of robust CAGEseq sites (potential TSS) in the region downstream of the *DACH1* first exon where *lncDACH1* would be expected.

## Reporting Summary

Further information on research design is available in the Nature Portfolio Reporting Summary linked to this article.

## Supplementary information

Reporting Summary

## Data Availability

No new data were generated for this work; RNAseq files from ENCODE were obtained for mouse heart development (https://www.encodeproject.org/organism-development-series/ENCSR452PJM/), aortic SMCs (https://www.encodeproject.org/experiments/ENCSR000AAA/), and coronary artery SMCs (https://www.encodeproject.org/experiments/ENCSR000AAG/). Quiesced saphenous vein SMC RNAseq files are available at GEO (GSE69637). FANTOM CAT transcript annotation and CAGEseq data were viewed at https://fantom.gsc.riken.jp/zenbu/gLyphs/index.html.
